# Three-dimensional conformal radiation for esophageal squamous cell carcinoma with involved-field irradiation may deliver considerable doses of incidental nodal irradiation

**DOI:** 10.1186/1748-717X-7-200

**Published:** 2012-11-27

**Authors:** Kai Ji, Lujun Zhao, Chengwen Yang, Maobin Meng, Ping Wang

**Affiliations:** 1 Department of Radiotherapy, Tianjin Medical University Cancer Institute and Hospital, and Tianjin Key Laboratory of Cancer Prevention and Therapy, Tianjin, PR China

**Keywords:** Esophageal squamous cell carcinoma, Radiotherapy, Incidental irradiation

## Abstract

**Background:**

To quantify the incidental irradiation dose to esophageal lymph node stations when irradiating T1-4N0M0 thoracic esophageal squamous cell carcinoma (ESCC) patients with a dose of 60 Gy/30f.

**Methods:**

Thirty-nine patients with medically inoperable T1–4N0M0 thoracic ESCC were treated with three-dimensional conformal radiation (3DCRT) with involved-field radiation (IFI). The conformal clinical target volume (CTV) was re-created using a 3-cm margin in the proximal and distal direction beyond the barium esophagogram, endoscopic examination and CT scan defined the gross tumor volume (GTV) and a 0.5-cm margin in the lateral and anteroposterior directions of the CT scan-defined GTV. The PTV encompassed 1-cm proximal and distal margins and 0.5-cm radial margin based on the CTV. Nodal regions were delineated using the Japanese Society for Esophageal Diseases (JSED) guidelines and an EORTC-ROG expert opinion. The equivalent uniform dose (EUD) and other dosimetric parameters were calculated for each nodal station. Nodal regions with a metastasis rate greater than 5% were considered a high-risk lymph node subgroup.

**Results:**

Under a 60 Gy dosage, the median D_mean_ and EUD was greater than 40 Gy in most high-risk nodal regions except for regions of 104, 106tb-R in upper-thoracic ESCC and 101, 104-R, 105, 106rec-L, 2, 3&7 in middle-thoracic ESCC and 107, 3&7 in lower-thoracic ESCC. In the regions with an EUD less than 40Gy, most incidental irradiation doses were significantly associated with esophageal tumor length and location.

**Conclusions:**

Lymph node stations near ESCC receive considerable incidental irradiation doses with involved-field irradiation that may contribute to the elimination of subclinical lesions.

## Introduction

Radiotherapy has been indicated as a definitive treatment for unresectable or medically inoperable tumors in ESCC patients. However, the boundaries of the clinical target volume (CTV) are not internationally defined. In RTOG 85–01 [1], the range of radiotherapy was from the supraclavicular region to the gastroesophageal junction. In a subsequent study, RTOG 94–05 [2], 5-cm proximal and distal margins and a 2-cm lateral margin from the lateral border of the gross tumor volume (GTV) were recommended. The supraclavicular nodes were included only when the tumor was located in the cervical esophageal area. Three-dimensional conformal radiation (3D-CRT) was used in RTOG 01–33 [3], and the CTV was defined as the GTV plus 3-cm margins of proximal and distal normal esophagus. For cervical primary lesions, bilateral cervical lymph nodal regions were included. The planning target volume (PTV) included up to a 2-cm margin around the CTV. Thus, the extent of elective nodal irradiation was reduced in the latter two recommendations. However, thus far there are no literatures reporting that reducing field could lead to poor outcomes.

In two retrospective studies that included over 1000 patients, ENI (elective nodal irradiation) for esophageal squamous cell carcinoma (ESCC) was suggested because a high lymph node metastasis rate was noted [4,5]. However, recent clinical trials employing three-dimensional conformal radiotherapy (3DCRT) without intentional elective nodal irradiation have shown the rate of isolate out-field nodal failure (which was defined as a recurrent esophageal lesion and regional nodes that occurred inside the PTV) was only 2-8% [6-8]. In one prospective randomized study [8], 102 patients with histologically or cytologically diagnosed squamous cell cervical or upper-thoracic esophageal carcinoma were randomly divided into involve-field irradiation (IFI) groups or ENI groups. After a median follow-up of 37 months, there was no statistically significant difference between the 2 groups in 3-year overall survival rate and 3-year local control rates. As reported in the literature, esophageal cancer is associated with multicentric disease or submucosal “skip” invasion because of the extensive and longitudinal interconnecting system of lymphatics. In some cases, greater than 8 cm of normal tissue can exist between the gross tumor and micro-metastatic skip areas secondary to this extensive lymphatic network [9]. This phenomenon is inconsistent with the low incidence of isolate out-field nodal failure reported in 3D-CRT studies. The reasons for the discrepancy between the risk of microscopic disease in surgical series and the low incidence of isolate out-field nodal failure in radiation series include but not limited to the following: (1) high incidences of local failure and distant metastases, which may mask regional nodal failure because many patients die before their regional nodal disease becomes clinically apparent, (2) patients undergoing radiation series often have severe co-morbidity such that there may not be enough time for micro-metastases to develop into a clinically detectable nodal failure, (3) untreated or inadequately treated micro-metastases in the out-field nodes may not result in clinical nodal failures and they may be sources of distant metastases later, and (4) micro-metastases may be adequately controlled by the incidental nodal irradiation.

The purpose of our study was to quantify the incidental irradiation doses to esophogeal lymph node stations when treating T1-4N0M0 ESCC patients with a dose of 60 Gy/30f. We also sought to analyze the feasibility of IFI using 3D-CRT for ESCC patients from the perspective of radiation dosimetry study to esophogeal lymph node stations.

## Materials and methods

### Patients and treatment plan

Thirty-nine patients with medically inoperable T1–4N0M0 thoracic ESCC were treated at our center from February 1, 2011 to May 1, 2012. All patients included in this study had available simulation CT scans. The clinical characteristics of the patients are shown in Table 1. This study was granted by Tianjin Medical University Cancer Institute and Hospital Ethics Committee. Simulation CT scanning was performed using helical CT and 3-mm slice thickness (CT Brilliance, Philips Medical Systems, The Netherlands) was performed using intravenous contrast. All patients were immobilized in a supine position (with thermoplastic on the chest) when simulation and radiotherapy was performed. The scanned area was from the angulusmandibulae to the bottom of the L1 vertebral body. These images were transferred to a 3-D planning system (ADAC Pinnacle^3^ 8.0m, Philips Medical Systems, USA). Because the actual CTV included elective nodal regions which were not clinically diagnosed with lymph node metastasis, the CTV in this study was re-created using a 3-cm margin in the proximal and distal direction (following the course of the esophagus) beyond the barium esophagogram, endoscopic examination and CT defined GTV, and a 0.5-cm margin in the lateral and anteroposterior directions of the CT scan-defined GTV. The PTV encompassed 1-cm proximal and distal margins and 0.5-cm radial margin, based on the CTV. Treatments were designed using computerized radiation dosimetry, and delivered by 6-MV X-rays from a linear accelerator (Elekta Precise Linear Accelerator, Sweden). The PTV was covered by at least 95% isodose surface and 95% of The PTV should receive the 60 Gy of prescribed dose. The maximum dose within the PTV was not allowed to exceed 110% of the prescribed dose. Each treatment plan consisted of a median of three static fields (range: 3–4) with the following normal tissue constraints: (1) the mean lung dose (MLD) was ≤13 Gy, V5 (*i.e.*, percentage of lung volume receiving ≥5 Gy) was ≤50%, V20 ≤25%, and V30 ≤20%, (2) the volume of heart receiving ≥40 Gy was ≤30%, and (3) the maximum spinal cord dose was ≤45 Gy. If these constraints could not be satisfied, the plan would be compromised, with an MLD of <15 Gy, lung V20 ≤30%, and ≤40% of the heart volume received ≥40 Gy.

**Table 1:** The clinical characteristics of thoracic ESCC patients (n=39)

**Location**	**Upper**	**Middle**	**Lower**
Number	12	15	12
Length (cm)	6.75 (3.0-9.9)	7.5 (4.5—12.0)	5.9 (2.0—9.0)
Volume (cm^3^)	17.9 (4.0-74.4)	25.8 (9.0—83.5)	15.5 (2.4—105.0)
T stage			
T1-3	8	11	10
T4	4	4	2

### Nodal station delineation and incidental radiation dose

To evaluate the dose of incidental irradiation each nodal region received, we delineated nodal regions using the Japanese Society for Esophageal Diseases (JSED) guidelines (Table 2) [10] and an EORTC-ROG expert opinion [11] with each patient. We delineated regions 3 and 7 together as region “3&7” because of difficulty distinguishing demarcation of regions 3 and 7 in some patients’ CT images. The mean dose (D_mean_) and the percentage of volume receiving more than 20 Gy (V20), 30 Gy (V30), 40 Gy (V40), and 50 Gy (V50) for each region were calculated. Due to the significantly heterogeneous dose distribution of some lymph nodal regions, the equivalent uniform dose (EUD) was also calculated for each contoured nodal region. EUD is the biologically equivalent dose that, if homogeneously given, would kill the same cells in the tumor volume as the actual non-uniform dose distribution. EUD was calculated using the following formula:

$$\;\text{EUD=}{\left(\frac{1}{N}\sum_{\text{i}}D_{i}^{a}\right)}^{\frac{1}{\text{a}}}$$

**Table 2:** Terminology of regional lymph node in esophageal carcinoma by JSED

**Numbering**	**Cervical and mediastinal lymph nodes**	**Numbering**	**Abdominal lymph nodes**
101	Cervical paraesophageal	1	Right cardial
104	Supraclavicular	2	Left cardial
105	Upper thoracic paraesophageal	3	Lesser curvature
106rec-L	Left recurrent nerve	7	Left gastric artery
106rec-R	Right recurrent nerve	8	Common hepatic artery
106pre	Pretracheal		
106tb-L	Left tracheobronchial		
106tb-R	Right tracheobronchial		
107	Subcarinal		
108	Middle thoracic paraesophageal		
109	Main bronchus lymph nodes		
110	Lower thoracic paraesophageal		
111	Supradiaphragmatic		
112ao	Thoracic paraaortic		
112pul	Pulmonary ligament		
113	Ligamentum arteriosum		
114	Anterior mediastinal		

JSED: Japanese Society for Esophageal Diseases.

N is the number of voxels in the anatomic structure of interest, D_i_ is the dose in the *i*th voxel, and ‘a’ is the tumor-specific parameter that can make cold spots of interest area reflected by the value of EUD when its value is less than 1. In this study, we choose ‘a’ as −5. The nodal region whose metastasis rate was greater than 5% was considered a high-risk lymph node subgroup [5]. The high-risk regions of upper-thoracic ESCC were 101, 104, 105, 106rec-L, 106tb-L, 106tb-R, and 107 (see Table 2 for definition of terminology). The regions 101, 104-R, 105, 106rec-L, 106tb-L, 106tb-R, 107, 108, 110, 112, 2, 3, and 7 were deemed high-risk regions in middle-thoracic ESCC. The high-risk regions of lower-thoracic ESCC were 107, 108, 110, 112, 1, 2, 3, and 7.

### Statistical analyses

The dose data were first evaluated for their distribution, and determined not to be gaussian distribution. Therefore, non-parametric correlation analyses (Spearman’s R) were used. Statistical significance was defined as a p value ≤0.05.

## Results

The dosimetric parameters of incidental irradiation of upper, middle, and lower thoracic ESCC patients are shown in Tables 3, 4, and 5. Under a prescribed 60 Gy dose, the median D_mean_ and EUD was greater than 40 Gy in most high-risk nodal regions.

**Table 3:** Dosimetric parameters of incidental irradiation in high-risk nodal regions for upper-thoracic ESCC [median mean dose (range)]

	**D**_**mean**_**(cGy)**	**EUD (cGy)**	**V20 (%)**	**V30 (%)**	**V40 (%)**	**V50 (%)**
101	6150.1(3188.1-6567.4)	6110.1(220.2-6553.6)	100(53–100)	100(48–100)	100(48–100)	99.5(42–100)
104	4169.4(2571.4-6675.2)	1517.6(207.7-6623.0)	94(56–100)	68(37–100)	50(28–100)	38.5(20–100)
105	6256.2(5744.0-6800.0)	5800.5(982.4-6518.2)	100(96–100)	100(91–100)	99(84–100)	97.5(81–100)
106rec-L	6342.0(6240.5-6599.8)	6337.2(6235.2-6585.6)	100(100–100)	100(100–100)	100(100–100)	100(100–100)
106tb-L	5637.6(4607.9-6198.8)	4734.5(964.7-5933.8)	100(88–100)	100(76–100)	86(62–100)	73(54–95)
106tb-R	4606.8(1869.0-6079.6)	3638.2(562.2-5930.7)	100(40–100)	98(21–100)	65(6–100)	40(0–99)
107	6225.7(297.0-6433.0)	6120.2(176.8-6409.2)	100(0–100)	100(0–100)	100(0–100)	100(0–100)

L: the left side; R: the right side.

**Table 4:** Dosimetric parameters of incidental irradiation in high-risk nodal regions for middle-thoracic ESCC [median mean dose (range)]

	**D**_**mean**_**(cGy)**	**EUD (cGy)**	**V20 (%)**	**V30 (%)**	**V40 (%)**	**V50 (%)**
101	122.3(77.9-2788.0)	94.2(60.2-427.6)	0(0–50)	0(0–41)	0(0–40)	0(0–30)
104-R	131.8(83.0-1269.4)	109.7(67.4-366.7)	0(0–26)	0(0–10)	0(0–5)	0(0–2)
105	4273.1(1854.8-6322.4)	338.7(135.0-5284.1)	72(29–100)	67(28–99)	65(27–96)	61(21–95)
106rec-L	3540.1(735.6-6400.23)	333.5(177.6-6387.4)	59(9–100)	53(9–100)	53(5–100)	52(0–100)
106tb-L	6033.3(4725.5-6412.5)	5793.1(3186.7-6402.5)	100(99–100)	100(92–100)	100(74–100)	95(44–100)
106tb-R	6043.8(4477.9-6466.4)	5484.6(3171.3-6350.2)	100(100–100)	100(76–100)	99(60–100)	87(42–100)
107	6297.0(6241.8-6457.5)	6295.1(6221.3-6456.5)	100(100–100)	100(100–100)	100(100–100)	100(100–100)
108	6341.2(6198.1-6430.1)	6337.9(6069.4-6426.3)	100(100–100)	100(100–100)	100(100–100)	100(100–100)
110	6128.2(1505.1-6525.8)	4364.6(220.2-6519.72)	100(27–100)	100(21–100)	99(20–100)	94(14–100)
112ao	5625.7(4056.7-6449.0)	3194.2(254.8-6367.2)	100(68–100)	99(67–100)	90(60–100)	73(56–99)
112pul-L	5763.5(4529.2-6402.3)	5572.0(2353.0-6401.4)	100(100–100)	100(94–100)	100(61–100)	91(33–100)
112pul-R	6166.0(5667.2-6516.1)	6134.6(4823.0-6515.5)	100(100–100)	100(99–100)	100(91–100)	100(85–100)
2	378.0(135.1-6409.3)	358.5(132.2-6396.5)	0(0–100)	0(0–100)	0(0–100)	0(0–100)
3&7	206.3(55.1-3213.6)	173.1(52.4-883.8)	0(0–67)	0(0–50)	0(0–35)	0(0–25)

L: the left side; R: the right side. * Lymph node from 8 to 16.

**Table 5:** Dosimetric parameters of incidental irradiation in high-risk nodal regions for lower-thoracic ESCC [median mean dose (range)]

	**D**_**mean**_**(cGy)**	**EUD (cGy)**	**V20 (%)**	**V30 (%)**	**V40 (%)**	**V50 (%)**
107	5500.9(391.1-6630.3)	3371.9(317.2-6626.6)	100(0–100)	92(0–100)	90(0–100)	85(0–100)
108	6216.6(3956.6-6533.2)	4694.9(354.9-6530.0)	100(65–100)	97(60–100)	97(59–100)	94.5(54–100)
110	6505.0(6344.5-6975.0)	6492.6(6329.3-6959.9)	100(100–100)	100(100–100)	100(100–100)	100(100–100)
112ao	5389.4(4211.3-6196.9)	2244.7(375.3-5540.3)	98(79–100)	96(78–100)	82(65–100)	69.5(50–96)
112pul-L	6207.9(2848.9-6796.4)	6163.5(1070.9-6791.2)	100(63–100)	100(43–100)	100(22–100)	100(14–100)
112pul-R	6318.8(2539.2-7197.1)	6313.8(2399.9-7193.1)	100(98–100)	100(11–100)	100(0–100)	100(0–100)
1	6038.7(304.0-6322.2)	6036.3(295.1-6321.8)	100(0–100)	100(0–100)	100(0–100)	100(0–100)
2	6429.9(3179.2-6960.7)	6401.6(1246.8-6958.3)	100(81–100)	100(52–100)	100(25–100)	100(0–100)
3&7	4407.2(342.7-6213.3)	2078.3(284.5-6199.3)	94(0–100)	84(0–100)	63(0–100)	43(0–100)

L: the left side; R: the right side.

**Figure 1:**
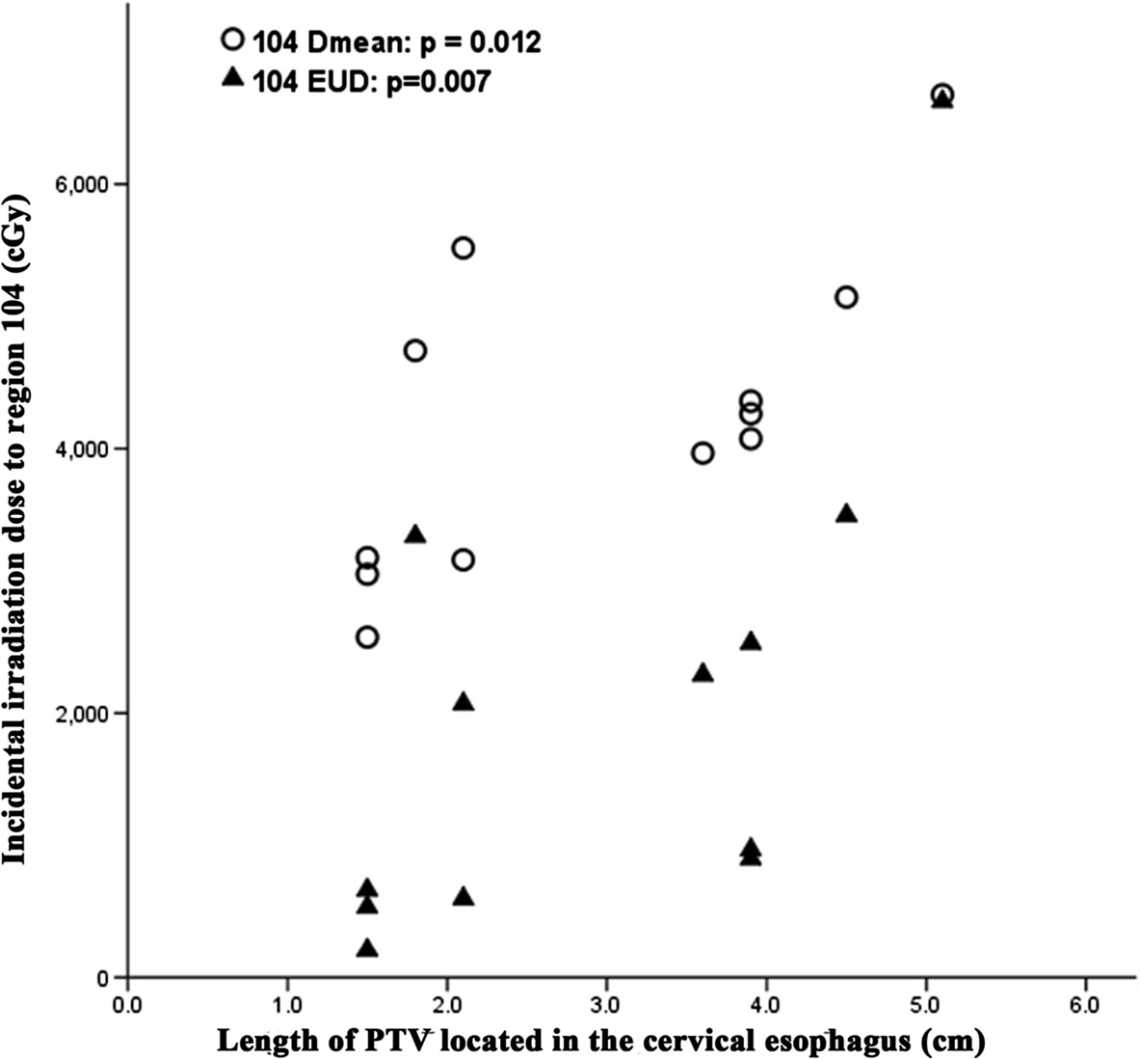
**Relationship between the PTV length located in the cervical esophagus and incidental irradiation dose to region 104 of upper-thoracic ESCC.** EUD, equivalent uniform dose.

**Figure 2:**
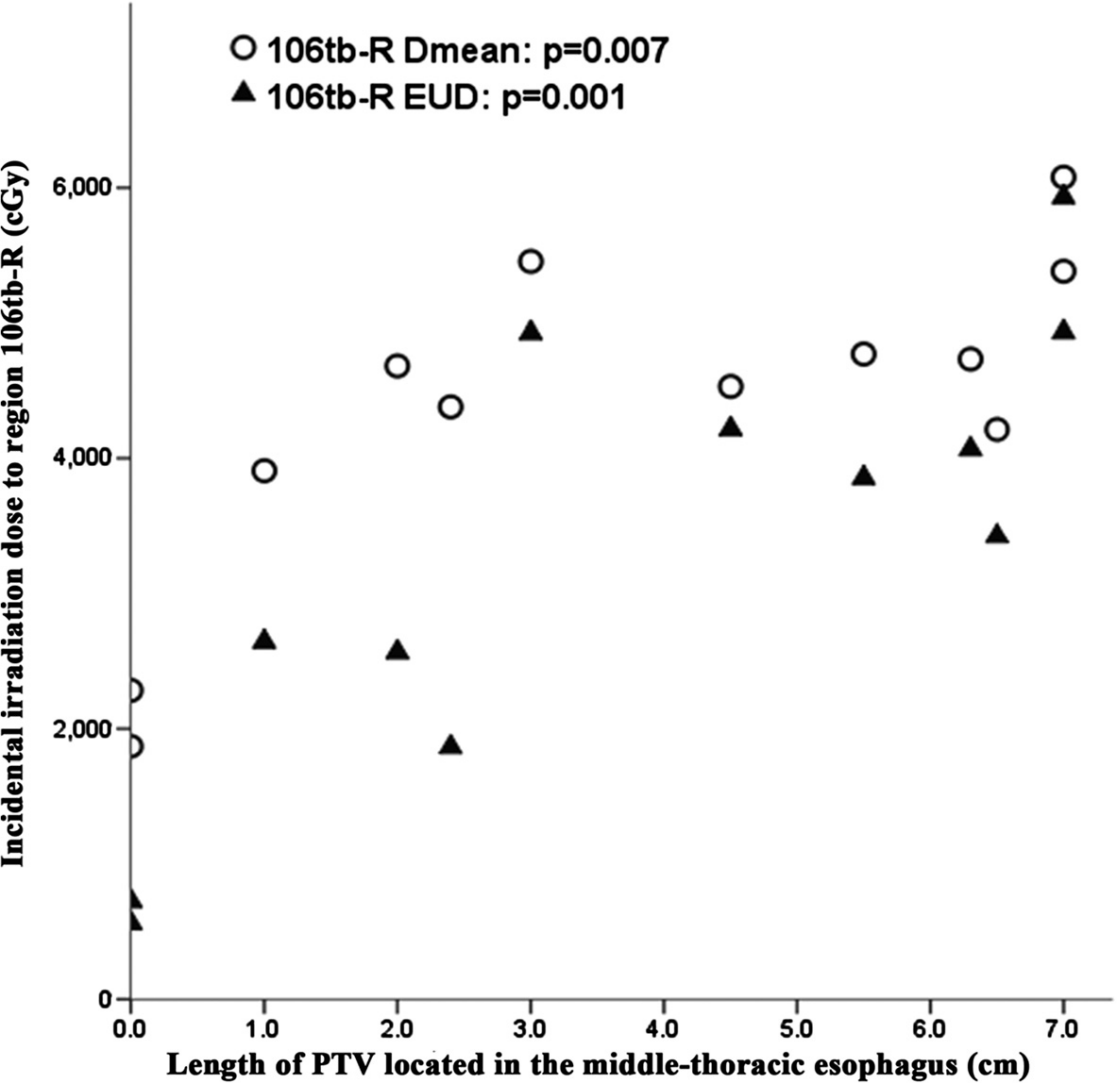
**Relationship between the PTV length located in the middle-thoracic esophagus and incidental irradiation dose to region 106tb-R of upper-thoracic ESCC.** EUD, equivalent uniform dose.

For the 104 and 106tb-R subgroups of high-risk nodal regions in upper-thoracic ESCC, the median EUD was only 1517.6 and 3638.2 cGy, although the median D_mean_ reached 4169.4 and 4606.8 cGy. However, the incidental irradiation dose of these two regions was significantly associated with the length and location of the esophageal tumor. For region 104, the incidental irradiation dose was significantly associated with the length of PTV located in the cervical esophagus (Figure 1) and the correlation coefficient (r) and P values of D_mean_ were 0.696 and 0.012, respectively. Similarly, for EUD of region 104, the r and p values were 0.732 and 0.007, respectively. In addition, the incidental irradiation dose of 106tb-R was significantly associated with the length of PTV located in the middle-thoracic esophagus (Figure 2). For D_mean_, the r and p values were 0.733 and 0.007, respectively. The values of r and p were 0.835 and 0.001 for EUD, respectively. In addition, V40 and V50 values of all high-risk nodal regions of upper-thoracic ESCC, except 104 and 106tb-R, were greater than 85% and 70%, respectively.

**Figure 3:**
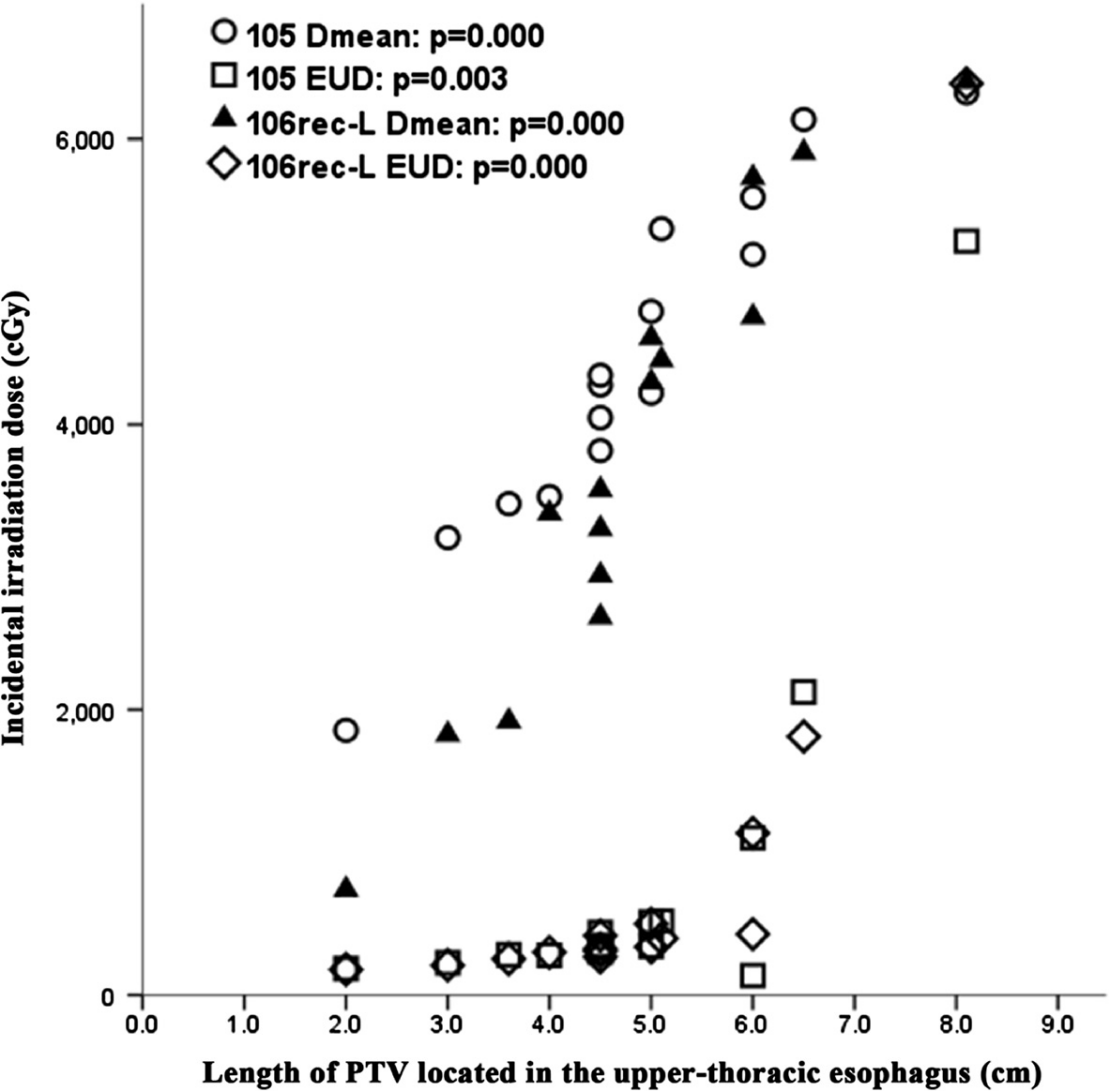
**Relationship between the PTV length located in the upper-thoracic esophagus and incidental irradiation dose to region 105 and 106rec-L of middle-thoracic ESCC.** EUD, equivalent uniform dose.

For high-risk nodal regions with an EUD less than 40 Gy of middle-thoracic ESCC, the incidental irradiation dose of 101, 104-R, 2, and 3&7 was so low that the dose in these regions could be ignored. Nevertheless, the incidental irradiation dose of 105 and 106rec-L of middle-thoracic ESCC was significantly associated with the length of PTV located in the upper-thoracic esophagus (Figure 3). For D_mean_, the r and P values were 0.962 and 0.000 for region 105, respectively, and 0.957 and 0.000 for region 106rec-L, respectively. For EUD, the r and P values were 0.706 and 0.003, and 0.937 and 0.000, respectively. This indicates that patients with longer lesions and with more of the tumor mass on top of the esophagus had a higher D_mean_ and EUD in region 105 and 106rec-L of middle-thoracic ESCC. Furthermore, except for regions of 101, 104-R, 105, 106rec-L, 2, and 3&7, the values of V40 and V50 for all high-risk nodal regions of middle-thoracic ESCC were not less than 90% and 80%, respectively.

**Figure 4:**
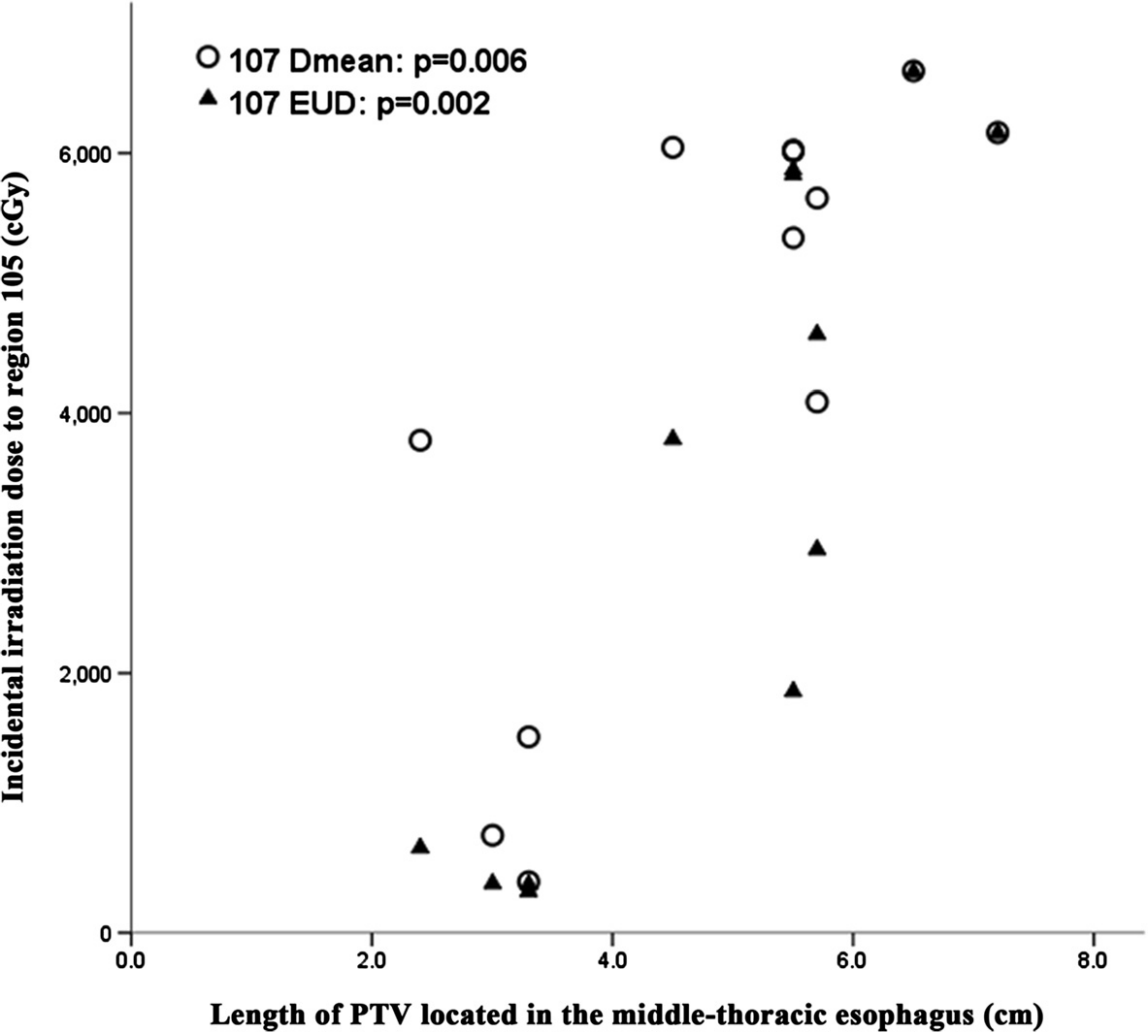
**Relationship between the PTV length located in the middle-thoracic esophagus and incidental irradiation dose to region 107 of lower-thoracic ESCC.** EUD, equivalent uniform dose.

Likewise, a significant correlation was seen between the length of PTV located in the middle-thoracic esophagus to the D_mean_ and EUD of region 107 in lower-thoracic ESCC (r = 0.735, p = 0.006; r = 0.802, p = 0.002) (Figure 4). However, no correlation was observed for the length of PTV located in the abdomen to incidental irradiation doses of region 3&7 (p = 0.124 and 0.103, respectively). In addition, V40 and V50 values of all high-risk nodal regions of lower-thoracic ESCC, except 3&7, were not less than 90% and 85%, respectively.

## Discussion

Currently, to the best of our knowledge, there have not been any studies regarding dosimetric evaluation of IFI for ESCC with 3D-CRT. This study demonstrated that the incidental irradiation dose to high-risk nodal regions is considerable. The incidental irradiation dose in most of the high-risk regions with an EUD less than 40 Gy was significantly associated with the length and location of the esophageal tumor. However, this association did not exist between the length of the PTV located in the abdomen and incidental irradiation dose of region 3&7 of lower-thoracic ESCC. Uncertainty of the stomach anatomy may explain the lack of correlation. Additionally, all high-risk nodal regions likely do not receive high enough incidental irradiation doses in all patients studied, even when the median D_mean_ or EUD was more than 40 Gy, a typical dose for ENI in most nodal regions. However, it has been reported that worthwhile treatment benefits can be achieved by lower doses (e.g., 10–30 Gy), and that a radiation dose as low as 24 Gy could reduce metastases by 30–50% [12-14].

It is generally accepted that the length of the thoracic esophageal lesion correlates with node metastasis. That is, the longer the thoracic ESCC, the greater the likelihood of lymph node metastasis [4,5]. In our study, incidental irradiation doses in regions 104 and 106tb-R of upper-thoracic ESCC, 105 and 106rec-L of middle-thoracic ESCC, and region 107 of lower-thoracic ESCC, were significantly associated with esophageal tumor length and location. This may explain why the isolate out-field nodal failure rate was low when employing 3DCRT without intentional elective nodal irradiation.

The isolate out-field nodal failure rate was low and overall survival did not decrease when IFI was used [6,7,15,16]. Zhao *et al.*[6] reported the results of a prospective study of 3D-CRT in 53 ESCC patients without distant metastases. Only the primary tumor and positive lymph nodes were irradiated in the study. Thirty-nine of the 53 patients (74%) showed treatment failure, but only three patients (8%) developed isolated out-of-field nodal recurrence. Button *et al.*[7] performed a retrospective study of 145 patients (75% with stage III-IV) of esophageal carcinoma (45% had adenocarcinoma) with conformal RT (50 Gy in 25 fractions). After RT, 85 patients (60%) had evidence of relapse at a median follow-up of 18 months, but only 3 patients (4%) developed relapse in regions adjacent to the RT fields. The low out-field failure maybe due to the incidental irradiation to elective nodal regions. In our study with T1-4N0M0 patients, most high-risk nodal regions receive considerable doses, especially patients with long lesions. In clinical practice, however, more patients were presented with positive lymph node metastasis, in which the incidental irradiation dose to high-risk regions would much higher. Additionally, more high-risk regions would receive considerable doses if metastatic nodes were included in GTV. As a result, we believe that incidental irradiation may play a role in the control of micro-metastasis.

In the study, the prescribed dose was 60Gy/30f by conventional fractionation. This dose considers the discrepancy of tumor radiosensitivity and tolerance of chemoradiotherapy between Western populations and Asian populations, despite the fact that patients do not obtain benefits with a higher dose than 50.4Gy/28f, according to RTOG 94–05 [2]. In our study, the CTV was generated with a 3 cm margin to the GTV. Incidental irradiation to the cranio-caudal direction outside the treatment field should be much lower. However, in the cranio-candal directions, a 3–5 cm margin is usually added to the GTV to generate CTV, and, after radiation therapy, lymph node recurrence at a region 3–5 cm far away from the GTV is not common [6-8,15,16]. Literatures also suggested that 3–5 cm margin in the cranio-candal direction can obtained similar results compared with larger margins [1-3].

In summary, our study demonstrated that, in thoracic esophageal carcinoma, involve-field irradiation may deliver considerable incidental dose to elective regions, which will have significant impact on the control of micro-metastasis. If incidental out-field nodal irradiation does control micro-metastasis, when use such modern radiation techniques as stereotactic radiotherapy, intensity modulated radiotherapy, and proton therapy, the omission of ENI should be performed with caution. Furthermore, the incidental irradiation dose is generally related to the conformal level of the plan, which is associated with the treatment technique, number of beams, beam arrangement, and leaf thickness of MLC. However, this study does not have the capability to investigate this question due to the relative uniform consideration of technical factors. Moreover, future clinical studies should be performed to evaluate the feasibility of IFI in the radiation therapy of esophageal squamous carcinoma.

## Abbreviations

ESCC: Esophageal squamous cell carcinoma; 3DCRT: Three-dimensional conformal radiation; IFI: Involved-field radiation; CTV: Clinical target volume; GTV: Gross tumor volume; PTV: Planning target volume; JSED: Japanese Society for Esophageal Diseases; EUD: Equivalent uniform dose; ENI: Elective nodal irradiation; MLD: Mean lung dose; D_mean_: Mean dose.

## Competing interests

The authors declare that they have no competing interests.

## Authors’ contributions

PW conceived the study and oversaw the study design and data collection; KJ and LZ helped to conceive and coordinate the study, to collect data, and to write the manuscript; KJ and CY delineated nodal regions and designed the treatment plans; MM, KJ and CY carried out the data and statistical analysis. All authors read and approved the final manuscript.
